# Supplementary material for: Uptake Rates of Risk-Reducing Surgeries for Women at Increased Risk of Hereditary Breast and Ovarian Cancer Applied to Cost-Effectiveness Analyses: A Scoping Systematic Review
Source: Cancers (Basel). 2022 Mar 31;14(7):1786. doi: 10.3390/cancers14071786 (PMC8997187; doi:10.3390/cancers14071786)
Supplement: Supplementary file 1 [file cancers-14-01786-s001.zip › cancers-1535390-supplementary.pdf]

Supplementary file

Uptake rates of risk-reducing surgeries for women at increased risk of hereditary breast and ovarian cancer applied to cost-effectiveness analyses

Julia Simões Corrêa Galendi [1]*, Sibylle Kautz-Freimuth[1], Stephanie Stock [1] and Dirk Müller [1,*]

Contents:

File S1. Search Strategy
Table S1. Input parameters for the German model included in the value of information analysis (reproduction from Müller et al
Table S2. Input parameters for the German model not included in the value of information analysis (reproduction from Müller et al
Table S3. Excluded studies

File S1. Search Strategy

Search term for literature search (PUBMED):

((costs and cost analysis[Mesh] OR decision support techniques*[Mesh] OR cost-effectiveness[TIAB] OR cost-utility[TIAB] OR cost-benefit[TIAB])) AND ((breast neoplasms[Mesh]) OR (ovarian neoplasms[Mesh]) OR (breast-ovarian[TIAB]) OR population-based[TIAB] OR (mammary cancer[TIAB]) OR (mammalian carcinoma)) AND (early detection of cancer[Mesh] OR genetic testing[Mesh] OR mutation*[TIAB] OR BRCA[TIAB] OR PALB2[TIAB] OR RAD51c/d[TIAB] OR TP53[TIAB] OR SNP[TIAB] OR ATM[TIAB])

Search term for literature search in the CRD Datebase, NHS EED, HTA, DARE and CEA Registry:

((breast cancer OR ovarian cancer OR BRCA) AND (cost effectiveness OR cost utility OR cost benefit) AND (genetic testing OR gene analysis OR multigene assay))

Table S1. Input parameters for the German model included in the value of information analysis (reproduction from Müller et al **[1]**).

Parameter Set	Input parameters	Value (SD)	Distributions
**Uptake rates**	Uptake of RRM	0.06 (0.02)	Beta
	Uptake of RRSO	0.42 (0.04)	Beta
	Uptake of both RRSO	0.45 (0.04)	Beta
	Uptake of intensified surveillance	0.07 (0.02)	Beta
**Incidence of cancer in *BRCA* carriers**	Incidence of Breast Cancer Carriers	35–39: 0.031 (0.002), 40–44: 0.021 (0.002), 45–49:0.023 (0.002), 50–54: 0.027 (0.002), ≥ 55: 0.033 (0.002)	Beta
	Incidence of Ovarian Cancer Carriers	0.039 (0.005)	Beta

	Incidence of contralateral breast cancer Carriers	0.067 (0.008)	beta
**Risk reduction due to preventive surgery**	Risk reduction of breast cancer after RRM	0.08 (0.01)	Log-normal
	Risk reduction of breast cancer after RRM and RRSO	0.05 (0.03)	Log-normal
	Risk reduction of ovarian cacner after RRSO	0.28 (0.01)	Log-normal
	Risk reduction of ovarian cancer after RRM and RRSO	0.14 (0.01)	Log-normal
	Risk reduction of contralateral breast cancer after RRM	0.05 (0.01)	Log-normal
	Risk reduction of contralateral breast cancer after RRM and RRSO	0.59 (0.02)	Log-normal
**Health related quality of life**	Health related quality of life in stage well	0.920 (0.002)	Beta
	Health related quality of life in stage well with positive test result	0.890 (0.02)	Beta
	Health related quality of life after RRSM	0.850 (0.24),	Beta
	Health related quality of life after RRSO	0.830 (0.24),	Beta
	Health related quality of life after RRM and RRSO	0.780 (0.25)	Beta
	Health related quality of life in stage breast cancer	0.679 (0.031)	Beta
	Health related quality of life in stage metastatics breast cancer	0.629 (0.045)	Beta
	Health related quality of life in stage ovarian cancer	0.52 (0.050)	Beta
	Health related quality of life in end stage ovarian cancer	0.160 (0.250)	Beta

Table S2. Input parameters for the German model not included in the value of information anaysis (reproduction from Müller et al **[1]**).

Parameter Set	Input parameters	Value (SD)	Distributions
**Other trasnistion probabilties**	Genetic testing positive	0.24 (0.003)	Beta
	Incidence of Breast Cancer non Carriers	35–39: 0.004 (0.001), 40–44: 0.007 (0.001), 45–49: 0.008 (0.001), 50–54: 0.012 (0.002), ≥ 55: 0 (0.001)	Beta
	Incidence of Ovarian Cancer non Carriers	0.002 (0.001)	Beta
	Incidence of contralateral breast cancer non Carriers	0.015 (0.003)	Beta
	Mortality of all causes	Age and gender specific	Beta
	Breast cancer mortality	35–39: 0.00 (0.000), 40–49: 0.001 (0.000), 50–59: 0.002 (0.000), 60–69: 0.004 (0.000),	Beta

		70–79: 0.007 (0.000), 80–89: 0.028 (0.001), 90–99: 0.139 (0.001), ≥ 100: 0.404 (0.002)	
	Ovarian cancer mortality	0.015 (0.005)	Beta
	Incidence ovarian cancer in previous breast cancer patients	0.015 (0.005)	Beta
	Probaility of developing metastasis in breast cancer patients	All ages: 0.0267 (0.0055)	Beta
**Cost parameters**	**Variable**	**Value in € (SD)**	
Diagnosis and monitoring			Gamma
	Ongoing high-risk screening / monitoring	560 (224)	Gamma
Surgical options			
	Prophylactic mastectomy<br>Prophylactic oophorectomy<br>Prophylactic mastectomy + oophorectomy<br>Prophylactic oophorectomy in case of BC<br>Prophylactic mastectomy in case of OC	8317 (3327)<br>2854 (1142)<br>11,171 (4468)<br>2854 (1142)<br>4783 (1913)	Gamma
	Therapeutic mastectomy	6556 (2622)	Gamma
	Breast-conserving surgery	4318 (1727)	Gamma
	Therapeutic oophorectomy	2854 (1142)	Gamma
Medication BC			
	Chemotherapy BC (year 1, proportion in hormone receptor subgroups)<br>Triple – (0.60)<br>Her2neu (0.10)<br>HR + (0.30)<br>Chemotherapy metastatic BC<br>Triple – (0.60)<br>Her2neu (0.10)<br>HR + (0.30)	 6371 (2548)<br>26,537 (10,615)<br>6371 (2548)<br>19,488 (7795)<br>48,775 (19,510)<br>12,201 (4880)	Gamma
	Endocrine therapy (Her2neu/HR)<br>BC, year 1/year 2-5<br>metastatic BC	1120 (448) / 320 (128) / 1120 (448)	Gamma
	Neutropenic sepsis	5782 (2313)	Gamma
	Neulasta (Pegfilgrastim)	9852 (3941)	Gamma
	Antiemetics	495 (198)	Gamma
	Bisphosphonates	421 (168)	Gamma
Other treatment BC			
	Adjuvant radiotherapy	1791 (716)	Gamma
	Local surgeries	8381 (3328)	Gamma

	Psychological treatment in case of cancer diagnosis [EBM, Consortium]	1231 (492)	Gamma
	Lymphatic drainage / physiotherapy (BC)	1480 (592)	Gamma
Medication OC			
	Not advanced<br>Advanced	10,387 (4155)<br>30,080 (12,032)	Gamma
	Recurrence (in advanced Ca)<br>Platin-resistent<br>Platin-sensitive	 5408 (2163)<br>30,080 (12,032)	Gamma
Palliative care			
	End of life treatment in metastatic BC or OC	11,145 (4458)	Gamma

Table S3. Excluded studies

Study/year	Main reason for exclusion
Balmana 2005 [2]	Risk reducing surgeries not considered
Gamble 2017 [3]	Populational testing
Patel 2018 [4]	Populational testing
Rubinstein 2009 [5]	Populational testing
Manchanda 2015 [6]	Risk reducing surgeries for sporadic cancer (not BRCA mutation carriers)
Manchanda 2016 [7]	Risk reducing surgeries for sporadic cancer (not BRCA mutation carriers)
Manchanda 2017 [8]	Populational testing
Manchanda 2018 [9]	Populational testing
Zhang 2019 [10]	Populational testing
Norum 2018 [11]	Insufficient reporting of the uptake rates of risk-reducing surgeries
Hoskins 2019 [12]	Choice of uptake rates was based on assumed perfect adherence
Tengs 2000 [13]	Choice of uptake rates was based on assumed perfect adherence

References

1. Muller, D.; Danner, M.; Schmutzler, R.; Engel, C.; Wassermann, K.; Stollenwerk, B.; Stock, S.; Rhiem, K. Economic modeling of risk-adapted screen-and-treat strategies in women at high risk for breast or ovarian cancer. *Eur. J. Health. Econ.* **2019**, *20*, 739–750, doi:10.1007/s10198-019-01038-1.
2. Balmana, J.; Sanz, J.; Bonfill, X.; Casado, A.; Rue, M.; Gich, I.; Diez, O.; Sabate, J.M.; Baiget, M.; Alonso, M.C. Genetic counseling program in familial breast cancer: analysis of its effectiveness, cost and cost-effectiveness ratio. *Int. J. Cancer* **2004**, *112*, 647–652, doi:10.1002/ijc.20458.
3. Gamble, C.; Havrilesky, L.J.; Myers, E.R.; Chino, J.P.; Hollenbeck, S.; Plichta, J.K.; Kelly Marcom, P.; Shelley Hwang, E.; Kauff, N.D.; Greenup, R.A. Cost Effectiveness of Risk-Reducing Mastectomy versus Surveillance in BRCA Mutation Carriers with a History of Ovarian Cancer. *Ann. Surg. Oncol.* **2017**, *24*, 3116–3123, doi:10.1245/s10434-017-5995-z.
4. Patel, S.; Legood, R.; Evans, D.G.; Turnbull, C.; Antoniou, A.C.; Menon, U.; Jacobs, I.; Manchanda, R. Cost effectiveness of population based BRCA1 founder mutation testing in Sephardi Jewish women. *Am. J. Obstet. Gynecol.* **2018**, *218*, 431 e431–431 e412, doi:10.1016/j.ajog.2017.12.221.
5. Rubinstein, W.S.; Jiang, H.; Dellefave, L.; Rademaker, A.W. Cost-effectiveness of population-based BRCA1/2 testing and ovarian cancer prevention for Ashkenazi Jews: a call for dialogue. *Genet. Med.* **2009**, *11*, 629–639, doi:10.1097/GIM.0b013e3181afd322.
6. Manchanda, R.; Legood, R.; Pearce, L.; Menon, U. Defining the risk threshold for risk reducing salpingo-oophorectomy for ovarian cancer prevention in low risk postmenopausal women. *Gynecologic Oncology* **2015**, *139*, 487–494, doi:10.1016/j.ygyno.2015.10.001.
7. Manchanda, R.; Legood, R.; Antoniou, A.C.; Gordeev, V.S.; Menon, U. Specifying the ovarian cancer risk threshold of 'premenopausal risk-reducing salpingo-oophorectomy' for ovarian cancer prevention: a cost-effectiveness analysis. *J. Med. Genet.* **2016**, *53*, 591–599, doi:10.1136/jmedgenet-2016-103800.

8. Manchanda, R.; Patel, S.; Antoniou, A.C.; Levy-Lahad, E.; Turnbull, C.; Evans, D.G.; Hopper, J.L.; Macinnis, R.J.; Menon, U.; Jacobs, I.; et al. Cost-effectiveness of population based BRCA testing with varying Ashkenazi Jewish ancestry. *Am. J. Obstet. Gynecol.* **2017**, *217*, 578.e571–578.e512, doi:10.1016/j.ajog.2017.06.038.
9. Manchanda, R.; Patel, S.; Gordeev, V.S.; Antoniou, A.C.; Smith, S.; Lee, A.; Hopper, J.L.; MacInnis, R.J.; Turnbull, C.; Ramus, S.J.; et al. Cost-effectiveness of Population-Based BRCA1, BRCA2, RAD51C, RAD51D, BRIP1, PALB2 Mutation Testing in Unselected General Population Women. *J. Natl. Cancer Inst.* **2018**, *110*, 714–725, doi:10.1093/jnci/djx265.
10. Zhang, L.; Bao, Y.; Riaz, M.; Tiller, J.; Liew, D.; Zhuang, X.; Amor, D.J.; Huq, A.; Petelin, L.; Nelson, M.; et al. Population genomic screening of all young adults in a health-care system: a cost-effectiveness analysis. *Genet. Med.* **2019**, *21*, 1958–1968, doi:10.1038/s41436-019-0457-6.
11. Norum, J.; Grindedal, E.M.; Heramb, C.; Karsrud, I.; Ariansen, S.L.; Undlien, D.E.; Schlichting, E.; Maehle, L. BRCA mutation carrier detection. A model-based cost-effectiveness analysis comparing the traditional family history approach and the testing of all patients with breast cancer. *ESMO Open* **2018**, *3*, e000328, doi:10.1136/esmoopen-2018-000328.
12. Hoskins, P.; Eccleston, A.; Hurry, M.; Dyer, M. Targeted surgical prevention of epithelial ovarian cancer is cost effective and saves money in BRCA mutation carrying family members of women with epithelial ovarian cancer. A Canadian model. *Gynecol. Oncol.* **2019**, *153*, 87–91, doi:10.1016/j.ygyno.2019.01.018.
13. Tengs, T.O.; Berry, D.A. The cost effectiveness of testing for the BRCA1 and BRCA2 breast-ovarian cancer susceptibility genes. . Availabe online: http://www.scopus.com/inward/record.url?scp=0033997009&partnerID=8YFLogxK (accessed on 2 June 2021).
